# Co-creating active communities: processes and outcomes of linking public rehabilitation programs with civic engagement for active living in a Danish municipality

**DOI:** 10.1186/s40900-023-00495-6

**Published:** 2023-09-14

**Authors:** Anders Blædel Gottlieb Hansen, Marie Lønberg Hansen, Sanja Golubovic, Paul Bloch, Janne Kunchel Lorenzen, Thomas Peter Almdal, Mathias Ried-Larsen, Ida Kær Thorsen

**Affiliations:** 1 Centre for Clinical Research and Prevention, The Intersectoral Prevention Laboratory, Bispebjerg and Frederiksberg Hospital, Hovedvejen, Entrance 5, Nordre Fasanvej 57, 2000 Frederiksberg, Denmark; 2 Centre for Physical Activity Research, Rigshospitalet, University of Copenhagen, Section 7641, Blegdamsvej 9, 2100 Copenhagen, Denmark; 3 Health Promotion Research, Steno Diabetes Center Copenhagen, Borgmester Ib Juuls Vej 83, 2730 Herlev, Denmark; 4 Steno Diabetes Center Sjaelland, Birkevænget 3, 4300 Holbæk, Denmark; 5 Department of Endocrinology, Rigshospitalet, University of Copenhagen, Section 7562, Blegdamsvej 9, 2100, Copenhagen, Denmark; 6 Department of Immunology and Microbiology, Faculty of Health Sciences, University of Copenhagen, Copenhagen, Denmark; 7 Department of Sports Science and Clinical Biomechanics, University of Southern Denmark, Odense, Denmark

**Keywords:** Patient and public involvement, Co-creation, Public rehabilitation, Physical activity, Community-based, Partnership, Civil society

## Abstract

**Background:**

Increased levels of physical activity are associated with beneficial health effects for people with type 2 diabetes, cardiovascular disease and/or severe obesity; however, transforming knowledge about these effects into action is challenging. The aim of this paper is to explore lessons learnt from a co-creation process in a partnership project involving local stakeholders, including citizens, and researchers. The purpose of the process was to link a public health care institution with civil society organisations in the local community to make it possible for citizens to continue to be physically active after ending their public rehabilitation. Secondarily, this paper aims to develop a conceptual model of the above process.

**Methods:**

The study constitutes the first part of Project Active Communities and was based on a partnership between three research institutions and a Danish rural municipality, involving municipal and civil society stakeholders and citizens with type 2 diabetes, cardiovascular disease and/or severe obesity in co-creation of concrete interventions for implementation. The co-creation process was divided into two tracks, one involving citizens (two workshops) and one involving municipal and civil society stakeholders (two workshops). The two tracks were concluded with a final workshop involving all stakeholders, including local politicians. Data sources are focus groups and bilateral meetings, workshop observations, and questionnaires.

**Results:**

Lessons learnt include the importance of having a flexible timeframe for the co-creation process; giving room for disagreements and matching of mutual expectations between stakeholders; the value of a coordinator in the municipality to achieve acceptance of the project; and the significance of engaging local politicians in the co-creation process to accommodate internal political agendas. We have developed a conceptual model for a co-creation process, where we outline and explain three distinct phases: stakeholder identification and description, co-creation, and prototyping. The model can be adapted and applied to other sectors and settings.

**Conclusions:**

This study documents lessons learnt in a co-creation process aiming to link a public health care institution with civil society organisations in the local community. Further, this study has specified productive co-creative processes and documented the various phases in a conceptual model.

**Supplementary Information:**

The online version contains supplementary material available at 10.1186/s40900-023-00495-6.

## Abstract (plain language summary)

It is well known that physical activity has health benefits for people with chronic diseases. In this study, our aim was to explore lessons learnt from a co-creation process and develop a model for others to apply. The study was based on a partnership between three research institutions and a Danish rural municipality, involving municipal and civil society stakeholders and citizens with type 2 diabetes, cardiovascular disease and/or severe obesity. During the study, the above-mentioned stakeholders were invited to five workshops, where interventions for linking a public health care institution and civil society organisations were co-created. The five co-creation workshops led to the identification of four interventions, linking public health care institutions and civil society organisations. Lessons learnt from this project, which can be used by others who wish to design and conduct a co-creative process with diverse stakeholders, include:

- the importance of having a flexible timeframe for the co-creation process, as delays can easily occur in the unpredictable process of co-creation
- giving room for disagreements and matching of mutual expectations between stakeholders, as a common understanding of each stakeholder’s motives is important for the success of the project
- the importance and value of a coordinator in the municipality to achieve acceptance of the project
- the significance of engaging local politicians in the co-creation process to take internal political agendas into consideration.

We conclude by identifying three phases—a stakeholder, a co-creation, and a prototyping phase—in a model for co-creation that may be adapted and used by others.

**Supplementary Information:**

The online version contains supplementary material available at 10.1186/s40900-023-00495-6.

## Background

Increased levels of physical activity are associated with improved prognosis, increased functioning, and a better quality of life for people with type 2 diabetes, cardiovascular disease and/or severe obesity [1]. Despite the knowledge of these individuals about the beneficial effects of being physically active, there is a gap related to transforming this knowledge into action in everyday life [﻿^2^, ^3^]. To help them increase their physical activity level, these persons may be offered time-limited public rehabilitation, but for various reasons and despite being motivated to continue, many fail to maintain their new active habits when the rehabilitation program ends [4]. The term rehabilitation is heterogeneously used in different contexts. In a Danish context, rehabilitation is often synonymous with lifestyle intervention and self-management, targeting individuals with chronic diseases. Project Active Communities seeks to link a public health care institution and civil society organisations to encourage citizens with type 2 diabetes, cardiovascular disease and/or severe obesity to continue to be physically active, following the completion of a public rehabilitation program.

A recent review of scientific literature questions the long-term effects (> 6 months) of rehabilitation among persons with type 2 diabetes points to the need for a focus on the transition out of rehabilitation programs in order to maintain physical activity levels [4]. One approach could be to invite those who have ended a rehabilitation program to engage in a structured and supportive exercise environment, e.g. local sports, exercise, or civil society organisations [5]. In Denmark, civil society organisations constitute what has been termed a sustainable social structure that potentially could secure long-term engagement in physical activity [4]. However, implementing interventions to change health behaviour and securing sustainability over an extended period of time are challenging [6]. It is recommended to develop interventions through co-creative processes with end-users rather than applying top-down approaches [7]. This may be achieved by supporting the local community in developing solutions that can realistically be implemented and are sustainable [8]. Working through partnerships makes it possible for several stakeholders to achieve a joint understanding of a specific problem and to find solutions together [9]. This approach is increasingly being advocated as a way to facilitate collaborative work within and across sectors [10]. We believe that mobilization and engagement of local stakeholders and increased awareness of the challenges and needs among citizens in the target group are important. These aspects are both preconditions and key mechanisms for creating sustainable change [11].

In a Danish context, several studies have been published with a focus on co-production in municipalities [12–15]. Previous studies have focused on collaboration between sports clubs and public institutions [15], challenges in organising mental health service between public professionals and civil society [13], institutional logics when linking public health care and civil society [14], and collaboration on activities for older adults between voluntary associations and municipalities [12]. To our knowledge no previous study has focused on the interplay between municipality, citizens, and civil society organisations when linking public health care institutions and civil society organisations in the local community. This study contributes with new insights by including the perspectives of citizens in the co-creation of solutions and initiatives. Building on data from focus group interviews, bilateral meetings, questionnaires, and workshop observations, this paper aims to explore lessons learnt from the co-creation process in a partnership project involving municipal stakeholders, civil society organisations, citizens, and researchers. Secondarily, this paper aims to develop a conceptual model for a co-creation process.

## Methods

### Project active communities

This study constitutes a part of Project Active Communities that was initiated to enable a stronger linking between a public health care institution and civil society organisations in the local community, so that more citizens with type 2 diabetes, cardiovascular disease and/or severe obesity may continue to be physically active when their rehabilitation program ends. Project Active Communities consists of two studies focusing on a) development of linking interventions, and b) evaluation of these interventions (clinicaltrials.gov identifier: NCT05493345). Here, we report on the development of linking interventions only. Project Active Communities is based on a partnership between three research institutions: the Centre for Physical Activity Research (CFAS), the Intersectoral Prevention Laboratory (TIPL), and Steno Diabetes Center Sjaelland (SDCS); and the municipality of Odsherred, all in Denmark. A project group consisting of persons from the four institutions was responsible for the project’s management and progress.

Project Active Communities is set in Odsherred municipality, which like all other municipalities in Denmark is obliged to offer health-related rehabilitation as part of Danish public health care [16]. In general, health care in Denmark takes place in two complementary public sectors: the regional, specialised hospital services and the primary health care system that includes the general practitioners and the municipalities. Each sector has separate obligations and is regulated by distinctive laws and regulations [16, 17]. Rehabilitation is typically initiated at the hospital or by the general practitioner and finalised in a rehabilitation centre in a municipality, generally offering time-limited (6–12 weeks) rehabilitation programs [18]. Some of these programs include an introduction to the beneficial effects of exercise and may provide practical experience with certain forms of exercise [5].

In rural communities in Denmark, more people are obese and less physically active than those living in urban municipalities [19]. Odsherred municipality is such a rural municipality that faces several public health challenges. It is characterised as a relatively poor and sparsely populated municipality with a population size of appr. 33,000 living in a geographical area of appr. 357 square kilometres [20]. In rural municipalities such as Odsherred, citizens are characterised by low socio-economic status, and health services are often spread over large geographical distances with sparse infrastructure. This can lead to special challenges with rehabilitation. Yet, when measured by the number of civil society organisations (20 civil society organisations per 1000 inhabitants), the local community in Odsherred municipality is strong and affluent [20].

### Study design

The approach in this study included co-creation that builds on the premise that new insights and ideas can be obtained by involving end-users, local stakeholders, and researchers together in a joint engagement [7, 11, 21]. We define co-creation as a process where end-users, local stakeholders, and researchers together develop interventions, which they then jointly strive to implement. This co-creation process was conducted through a series of workshops, involving both citizens in the target group, municipal employees, and representatives from civil society in a common exploration of prevailing challenges and a search for possible solutions. We chose this approach because utilising stakeholders in the co-creation of public health interventions is thought to increase adherence and result in more effective, localised and sustainable solutions by tailoring interventions for specific groups and settings [7, 21–23]. In a following study, not reported here, the feasibility and fidelity of the co-created solutions will be evaluated to determine whether these solutions feed into the development of a scalable model for better linking public health care institutions with civil society organisations in the local community.

### Program theory

**Fig. 1:**
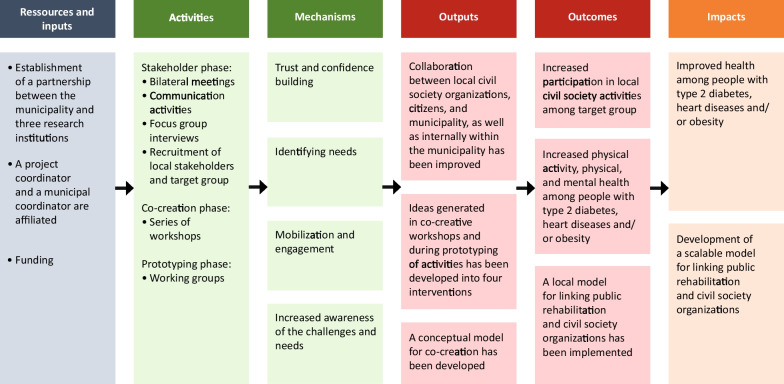
Program theory

A program theory for Project Active Communities was produced, iteratively revised, and used by the project group as a guiding tool for the project design. A simplified version of the program theory is shown in Fig. 1. The program theory can be seen as a roadmap showing how the project is expected to unfold and how resources and activities trigger certain mechanisms that, in a logical sequence, lead to the project’s outputs, outcomes, and impacts. In the present study, focus is on resources, activities, mechanisms, and outputs, whereas outcomes and impacts will be considered in the forthcoming evaluation study.

### Partnership process

**Fig. 2:**
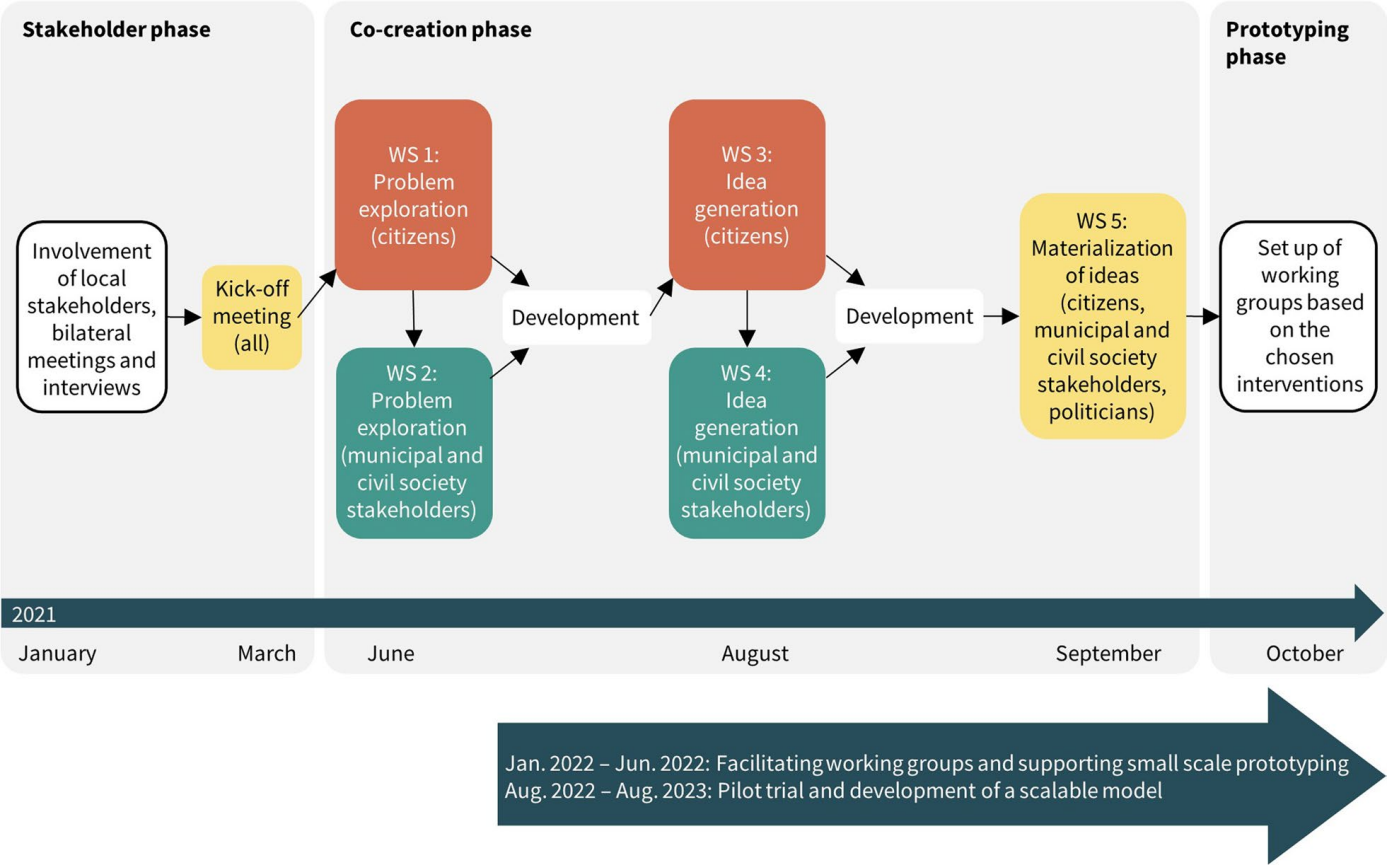
Depiction of the stakeholder, co-creation, and prototyping phases. *WS* workshop

CFAS and TIPL agreed on a preliminary project design that builds on a partnership approach and entails a series of co-creation workshops (Fig. 2). Subsequently, SDCS was contacted and agreed to be part of the partnership and they established the contact to Odsherred municipality. A partnership description was agreed upon and a project steering committee with a senior representative of each of the partners was established. Later in the process, a member from civil society was involved in the project steering committee, as this was requested by participants in one of the workshops. The research design was approved by all partners. CFAS provided the project management and TIPL contributed with the design of the co-creative process.

### Stakeholders

After forming the partnership, the identification of relevant local stakeholders was initiated. Overall, this process included problem scoping to identify municipal stakeholders, then mapping of local stakeholders including civil society organisations and citizens in the target group (i.e., persons with type 2 diabetes, cardiovascular disease and/or severe obesity), and concurrently having bilateral meetings with all potential stakeholders. The process was supported by a municipal project coordinator who was designated for approximately ten hours per week for six months to coordinate both between Odsherred municipality and CFAS and internally in the municipality. Furthermore, this person had the task of mobilising lead municipal administrators and politicians for the intersectoral action necessary for the project to succeed and to engage a municipal consultant on civil society organisations, who supported the mapping of local stakeholders. The project leader held a series of focus group interviews with citizens in the target group and bilateral meetings with heads of relevant municipal departments and their senior consultants. Several communication strategies were applied to engage local stakeholders, including involvement of the local media, establishment of a news mail, a website, and a Facebook page. This enhanced the project’s visibility in the local community. Overall, this preparatory work contributed to explain the rationale behind the project to the local stakeholders, to explore the attitudes to the partnership project and to engage stakeholders. Finally, all potential stakeholders were assembled to a kick-off meeting where the project was presented.

### Workshop participants

Based on the problem scoping process in the municipality, mapping of local stakeholders, and concurrent bilateral meetings, potential workshop participants were identified and supported by the municipal project coordinator and the municipal consultant on civil society organisations, the actual recruitment process for the workshops could start. Five citizens were recruited by the project leader among those participating in the focus group interviews (see Data sources subsection) and seven citizens were recruited from ongoing rehabilitation programs by a municipal health professional. Local sports, exercise, and civil society organisations were invited by an email from the municipal consultant on civil society organisation, who together with the project leader secured that the representatives had a wide geographical, sports type, and disease group representation. Based on follow-up bilateral meetings, the project leader recruited relevant organisation representatives. Municipal stakeholders (key employees and lead administrators) were recruited by the project leader in close collaboration with the municipal head of Odsherred health centre, and local politicians were recruited by municipal leaders. An overview of the workshop participants is presented in Table 1.

**Table 1:** Overview of workshop participants

	WS 1	WS 2	WS 3	WS 4	WS 5
Citizens (n)	12	4	10	3	8
Municipal leaders (n)	0	0	0	2	2
Municipal employees (n)	0	7	0	6	5
Patient association representatives (n)	0	3	0	2	3
Sports organisation representatives (n)	0	13	0	17	10
Municipal politicians (n)	0	0	0	0	4
Total (n)	12	27	10	30	32

*WS* workshop, *n* number of participants

WS 1: Participants were citizens with type 2 diabetes (n = 2), cardiovascular disease (n = 7), and severe obesity (n = 3).

WS 2: Participants were citizens with type 2 diabetes (n = 1) and cardiovascular disease (n = 3); municipal employees were physiotherapists (n = 3), a nurse (n = 1), a dietitian (n = 1), a consultant on civil society organisations (n = 1), and a team leader (n = 1); patient associations representing type 2 diabetes (n = 1), cardiovascular diseases (n = 1) and lung diseases (n = 1); and sports organisation representatives from gymnastics associations (n = 4), fitness associations (n = 4), a swimming association (n = 1), a multi-sport association (n = 2), and a local urban renewal association (n = 2).

WS 3: Participants were citizens with type 2 diabetes (n = 2), cardiovascular disease (n = 6), and severe obesity (n = 2).

WS 4: Participants were citizens with type 2 diabetes (n = 1) and cardiovascular disease (n = 2); municipal leaders were the head of Odsherred Health Centre and the head of “Department of Culture and Citizen”; municipal employees were physiotherapists (n = 2), nurses (n = 2), a consultant on civil society organisations (n = 1), and a team leader (n = 1); patient associations representing type 2 diabetes (n = 1) and lung diseases (n = 1); and sports organisation representatives from gymnastic associations (n = 6), fitness associations (n = 3), a multi-sport association (n = 2), a soccer association (n = 1), a golf club (n = 1), a regional sports association (n = 1), an adult education association (n = 1), and a local urban renewal association (n = 2).

WS 5: Participants were citizens with type 2 diabetes (n = 2), cardiovascular disease (n = 5), and severe obesity (n = 1); municipal leaders were the head of Odsherred Health Centre and the head of “Department of Health and Care” (n = 2); municipal employees were a physiotherapist (n = 1), nurses (n = 2), a dietitian (n = 1), and a consultant on civil society organisations (n = 1); patient associations representing type 2 diabetes (n = 1), cardiovascular diseases (n = 1), and lung diseases (n = 1); sports organisation representatives from gymnastic associations (n = 2), fitness associations (n = 3), a multi-sport association (n = 1), a soccer association (n = 1), a golf club (n = 1), a regional sports association (n = 1), and a local urban renewal association (n = 1); and municipal politicians were from the Culture and Leisure Committee and the Health and Care Committee (n = 4).

Moreover, researchers and project staff from the four partners were present at the workshops: WS 1, n = 5; WS 2, n = 7; WS 3, n = 5; WS 4, n = 8; and WS 5, n = 8.

In general, most participants attended all three workshops; however, some discontinued their participation, while others joined along the way.

### Data sources

Our approach to data collection and analysis was not a priori guided by a specific theoretical qualitative approach. Rather, we sought inspiration in frameworks for developing complex interventions [22, 24], and frameworks that combine qualitative research and patient and public involvement [25]. In these frameworks, an iterative and dynamic approach is a key principle, where interventions are developed by moving dynamically backwards and forwards between phases of overlapping actions, e.g. reviewing evidence, drawing on existing theory, and working with stakeholders in iterative cycles [22, 24, 25]. This process was reflected in our work where data were collected from different sources in the stakeholder (bilateral meetings) and co-creation phase. These data were combined with the stakeholders’ input from the workshops (workshops observations) to gain an in-depth understanding of their views and experiences, and collectively, this information was used between workshops by the project group to dynamically plan for the next workshop (depicted as “Development” in Fig. 2), and thereby drive the process of co-creation. Questionnaires were used for evaluating the process.

#### Focus group interviews

Prior to starting the co-creation phase, the CFAS project leader conducted three focus group interviews with citizens in the target groups who had partaken in a rehabilitation program. Interview participants were sampled by criterion-based purposeful sampling to select citizens representing engagement in civil society organisations (active/not active) and were recruited by a municipal health professional. Participants in the three focus group interviews were individuals with type 2 diabetes (n = 3), cardiovascular disease (n = 7) and severe obesity (n = 3). The interviews were semi-structured and contributed to the problem scoping process. A focus group schedule was developed, focusing on the challenges and difficulties for this group of citizens to stay physically active after finishing their rehabilitation program. Data were analysed by text condensation and the obtained understanding was used in the planning of content and focus of the subsequent co-creation workshops [26].

#### Bilateral meetings

As part of the problem scoping process, bilateral meetings were undertaken. The project leader met with senior public administrators in the municipality (n = 6). These meetings served the purpose of a) engaging in an informal dialogue about the core functions and activities of their departments and about the objectives and approach of the project, including its focus on the health benefits of physical activity, b) allowing department heads to express their perceptions about the project and whether (and how) they found it relevant to the core functions of their departments, and c) inviting department heads to participate in the co-creation process.

The project leader also engaged in approx. five bilateral meetings with representatives from relevant civil society organisations. The purpose of these meetings was to create a dialogue with the organisations regarding their resources and readiness to engage in the project, and their motives for and former experiences from collaborating with the municipality.

#### Workshop observations

To further document the co-creative process, workshop observations were written down by a neutral notetaker, collated and shared among the project group. Workshop observations consisted of a detailed report from the workshop including the plan for the workshop, characteristics of the participants, the process, the outcomes, and reflections from interactions in the groups. This information fed into the design of the next workshop.

#### Questionnaires

Questionnaires were developed to gauge the participants’ satisfaction with the workshop and consisted of statements ranked on a 5-point scale from strongly disagree to strongly agree, e.g.: (1) “I think the project is relevant”, (2) “I feel that my voice has been heard”, and (3) “I am motivated to continue participation”. The questionnaires were distributed and completed in paper form immediately after termination of each workshop.

#### Co‐creation method

In this study, we used a method of co-creation inspired by action research entitled “the Future Workshop” [7, 27, 28]. In accordance with “the Future Workshop”, participants were involved in a three-step process of (1) identifying key challenges, (2) generating ideas for action, and (3) transforming the ideas into concrete solutions [28]. We also used the “Framework for intervention co-production and prototyping” [22] that focuses on a combination of stakeholder consultations, an iterative process of co-production, and prototyping [22]. This framework was used to prototype several preliminary interventions that could be tested in rapid iteration and at a small scale. Rather than testing a few theory-based interventions, prototyping builds on design thinking, according to which ideas should be made tangible and real for target communities. This is considered an effective way to involve local communities [29]. These approaches were our sources of inspiration and were used selectively and rather pragmatically to fit into the context of the study. Overall, we wanted to understand the local practice, engage and involve local stakeholders in a dialogue about their needs and wishes, generate new ideas and practices, and define concrete and implementable solutions that later on could be tested and evaluated [7, 22, 28, 29]. The co-creation phase was divided into two tracks: one track for the citizens (two workshops) and one track for the municipal and civil society stakeholders (two workshops). These tracks joined in a final workshop for all stakeholders, including local politicians. The two parallel tracks were deliberately designed so that citizens in the target group, and municipal and civil society stakeholders could be heard separately, and so that outcomes from the citizens’ workshops could be presented in the workshops for municipal and civil society stakeholders. This presentation was facilitated by inviting representatives from the citizens’ workshop to participate in the workshops for the municipal and civil society stakeholders (see Fig. 2).

**Fig. 3:**
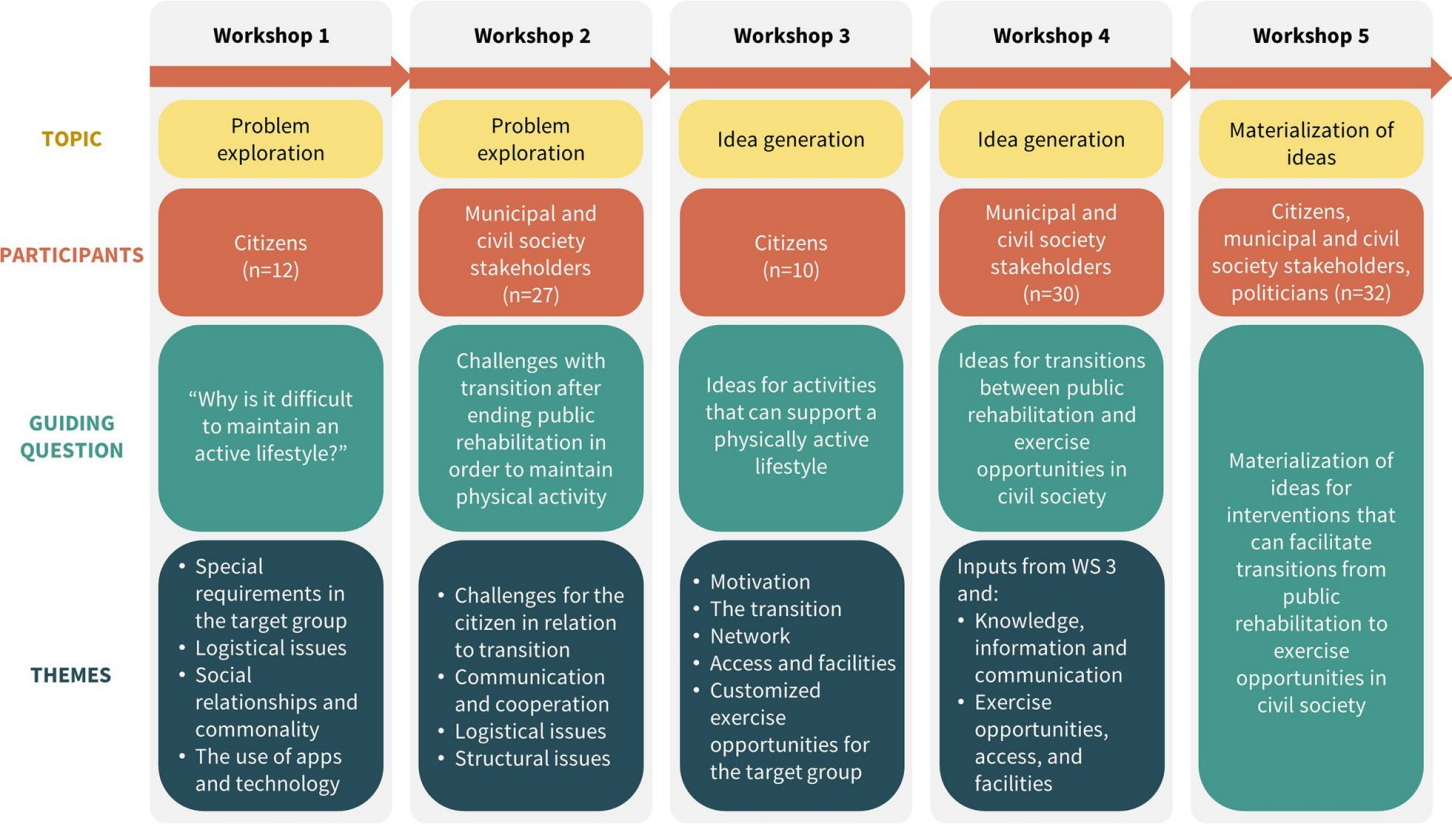
Topic, participants, guiding question, and themes of the five workshops. *WS* workshop

Five co-creation workshops took place from June to September 2021. The workshops were led by the CFAS project leader, facilitated by an experienced facilitator (PB co-author), and supported by TIPL, SDCS and CFAS. Facilitation tools and techniques were decided upon among the project group. The format, venue and timeframe (three hours) were the same for all workshops and built around a shared introduction to the project, facilitated group work, a meal and a shared conclusion and introduction to the next workshop. Between the workshops, development work was done by the project group: Inputs from the workshops were collated, based on which themes, agendas, and facilitation scripts for the following workshops were finalised. Figure 3 provides an overview of the topics, participants, guiding questions, and themes of the five workshops.

Workshop 1 focused on problem exploration and exclusively included citizens with type 2 diabetes, cardiovascular disease and/or severe obesity and experience with public rehabilitation. Participants were divided into three smaller groups, where they could discuss the challenges, they experienced in maintaining a physically active lifestyle. Group discussions were guided by a facilitator and used a dialogue tool, consisting of a series of pictures of everyday situations, to inspire the participants to an open and free reflection [30]. The discussions were then guided by four themes (see Fig. 3). During the break, the project group condensed the citizens' inputs into concrete topics. After the break, all citizens ranked the topics according to what they experienced as the most significant challenges. The problems identified, now ranked, were presented in plenum, where citizens had the opportunity to qualify the inputs.

Workshop 2 also focused on problem exploration but this time among stakeholders from the municipality and civil society. Participants were split into two groups: One group containing municipal stakeholders and one group with stakeholders from civil society. In each group, representatives from workshop 1 were present. At the end of the group work, the identified problems were ranked by plenary voting as described in workshop 1.

Workshop 3 and workshop 4 included the citizens and the stakeholders from the municipality and civil society, respectively. Both workshops were built on the problems identified in workshop 1 and 2 and focused on idea generation. To involve and engage the participants in idea generation, workshop 3 was organised as a café event. The citizens chose a theme to be discussed in groups, facilitated by a project group member. Each group discussed at theme for 20 min and then rotated to a new theme. During the break, the project group condensed the citizens' inputs to topics. The solutions proposed for each theme were placed on a board. Citizens qualified the inputs along the way, but the ideas were not ranked.

In workshop 4, the participants were initially presented with the list of ideas that the citizens had identified in workshop 3 and with the challenges they themselves had identified during the problem exploration in workshop 2. These challenges were divided in two themes by the project group (see Fig. 3). The participants were divided into four groups, each including a facilitator from the project group. Each group discussed each theme for 25 min, and then rotated to a new theme. After the break, all inputs under each theme were presented, and time was set aside to qualify the inputs. The participants were given four votes and subsequently asked to rank the ideas.

The final workshop 5 included the citizens and stakeholders from the civil society and the municipality (including politicians). The focus was on materialising ideas and wishes into interventions that may facilitate transition from public rehabilitation to physical activities in the local community. In this workshop, the stakeholders could choose which of the four themes developed in workshop 4, they wished to discuss. Thus, discussions took place in four different groups where a facilitator guided a process consisting of filling out a predefined template with the purpose of making the proposed ideas more tangible and concrete. The completed templates were presented in plenum.

## Results

### Co-creation process

Workshop 1 resulted in four main outcomes: (1) Lack of motivation to get out of the door on your own, (2) Lack of guided support in the transition from rehabilitation to physical activities in the local community, (3) Lack of support from family and social network, and (4) Lack of exercise facilities to support and encourage physical activity.

In workshop 2, the highest ranked outcomes by municipal and civil society stakeholders were: (1) Lack of mutual matching of expectations, (2) Lack of knowledge about physical activities in the local community, (3) Lack of individualised support, (4) Lack of a link worker employed in the municipality, and (5) Lack of interdisciplinary dialogue.

The outcomes of workshop 3 were in the form of ideas and wishes: (1) A municipal employee as a link worker, (2) Citizen-driven supportive networks to encourage motivation, (3) Knowledge, interaction and trust between the municipality and civil society organisations, and (4) A wider palette of physical activities in the local community tailored to the citizens and the possibility for free (or reduced-price) physical activities.

Outcomes from workshop 4 in the form of ideas and wishes were: (1) United municipal effort to motivate citizens in the target group to use physical activities in the local community. (2) Digital portal for information about physical activities in the local community. (3) Volunteer centre that collects knowledge about physical activities in the local community. (4) A link worker to support transition out of rehabilitation and into civil society organisations.

The outcomes of workshop 5 were four broad potential interventions: (1) The need for developing a digital platform (in the form of a webpage) to enable strengthened information and knowledge sharing about exercise activities in the municipality (undefined target group); (2) The need for co-created activities across civil society organisations, targeting the special needs of the citizens, including feeling safe and welcome in a new environment; (3) The need for a volunteer centre to promote knowledge exchange between public rehabilitation and civil society and utilization of existing sport facilities; and (4) The need for strengthened municipal efforts and internal communication, with a request to employ a link worker in the municipality, functioning as a contact person between the municipality and the local sports, exercise, and civil society organisations.

Results from the post-workshop evaluation questionnaires showed that, in general, participants found the project relevant, felt that their voice had been heard, and most were motivated to continue participating in the process. This was most pronounced among the citizens in workshop 1 and workshop 3, where all but one strongly agreed in all statements, whereas in workshop 2—and to some extend workshop 4 and workshop 5—participants did not agree as strongly with the statements as in the remaining workshops. The participants' satisfaction with the five workshops can be seen in Additional file 1﻿.

### Prototyping of interventions

In line with Hawkins et al., we conceptualised the co-creation process as starting with stakeholder consultations, continuing to a series of co-creation workshops, and ending in a process of prototyping [22]. At the outset, we had the expectation that workshop 5 would result in several preliminary interventions that could be developed into testable prototypes. But the level of concretisation of the proposed interventions required more elaboration. Thus, over the subsequent four months, a process was organised whereby participants from the workshops—including citizens, municipal and civil society stakeholders—together with the project leader and other relevant stakeholders from the local community continued the co-creation process and further developed, refined, and clarified the content of the four interventions. This was done in working groups and at networking and dialogue meetings. When needed, experts and specialists were also involved. Based on each of the outcomes from workshop 5, the following interventions were developed: (1) a digital platform for health professionals and citizens in the target group; (2) co-created physical activities in the local community tailored to the needs of citizens; (3) creating a visiting program to integrate activities between local sports, exercise and civil society organisations, the municipality, and the citizens; and (4) upgrading municipal resources to achieve better transitions between public rehabilitation and physical activities in the local community by employing a link worker. The four interventions are expanded in Additional file 2.﻿ The project contributed seed funding (totalling 47,000 €) to the small-scale testing of local activities suggested in the workshops. The funds were, e.g., allocated to courses and educations, interventions to promote the recruiting and retention of members in local sports, exercise, and civil society organizations, and equipment for physical activities.

### Lessons learnt

Several important learnings arose from the project. The plan for the co-creation process had to be revised in regard to the content and format of the workshops when compared to what we had in mind from the start. Originally, we planned for three workshops, but decided to expand the co-creative process with two parallel tracks, one for the citizens in the target group and one for municipal and civil society stakeholders. This was due to potential challenges with power relations when, in the same workshop, both health professionals from the municipality and vulnerable citizens from the target group were in the problem exploration and idea generation phase. Furthermore, prototyping of interventions seemed rather difficult to realise after the five workshops and we had to expand the co-creation process with four working groups. One of the purposes of bringing people together in the workshops was to give the participants a voice and create a shared understanding. It turned out to be important to take old disagreements between stakeholders seriously and openly give space by bringing disagreements to the table and discuss them. Some of the disagreements were imagined and others were agreed upon by all actors. In line with this, lack of mutual matching of expectations was both a key finding and a surprise to us. We believe that a common understanding of each stakeholder’s motives was important for the success of the project.

A key lesson learnt about the practice of co-creation from this project was the value of a full-time project leader in the research institution and a part-time project coordinator in the municipality. These persons turned out to be important links both between the municipality and the researchers and between the many stakeholders involved in the project. Furthermore, they contributed to creating trustful and good working relations between the municipality and civil society stakeholders, which promoted the communities’ acceptance of the project. Important competencies of the project leader were good communication skills, and being structured, enterprising and creative, whereas important competencies of the municipal project coordinator included being thorough, e.g., regarding information dissemination, and aligned with the municipal leaders. It proved to be of great value that the project leader from the research institution (CFAS) had several meetings and visits in the local community in the months leading up to the co-creation workshops. This contributed to increased awareness of and support to the project and was a help in recruiting participants to the workshops.

In the project group, we were aware of the significance of internal political processes in the municipality. Therefore, we engaged local politicians from different committees in the project and invited them to take part in the last co-creative workshop (workshop 5). After the workshop, we approached the heads of municipal administrations, and an online workshop was conducted to discuss and define success criteria that would commit them to financially support continued linking between the public health care institution and the civil society organisations in the local community. Endorsement of the project from the local politicians was of great significance in terms of general collaboration and agenda setting, related to the project. As a result, Project Active Communities was mentioned in the plan of action for realisation of the health policy in Odsherred municipality.

### Developing a conceptual model of co-creation

We aimed to develop a conceptual model of our co-creation process, as depicted in Fig. 2. The model is conceptualised as a procedure for involving the local community and creating the link between public health care institutions and civil society organisations. The overall focus is on co-creation between stakeholders and consists of three phases. In the first phase, the focus is on creating broad ownership to the project and an understanding that the issue of staying physical active after public rehabilitation is a problem that needs to be explored across different sectors and solved in cooperation with both citizens in the target group, civil society organisations, and the municipality (stakeholder phase). In the second phase, the focus was on engaging relevant, local stakeholders to develop concrete interventions for solving the problem (co-creation phase). In the third phase, interventions were developed and implemented together with administrators in the municipality and with local stakeholders (prototyping phase).

## Discussion

This study has identified lessons learnt in a project focussing on a process of co-creation between citizens and municipal and civil society stakeholders in a rural Danish municipality. Through a partnership between the municipality and three research institutions, a range of preparatory activities were conducted together with stakeholders from the local community. The challenge associated with the lack of sufficient linking between the public health care institution and the civil society organisations in the local community to support citizens in the target group in maintaining physical activity levels resonated well with challenges perceived by the municipality and civil society. The combination of a thorough problem exploration in the local community, a process inspired by action research, and a framework for co-creation, resulted in local engagement and mobilization of central stakeholders. In the workshops, the different perspectives of both the citizens in the target group and the municipal and civil society stakeholders were discussed. Through a facilitated process of co-creation that gave rise to a common understanding of the challenges related to linking the public health care institutions with the civil society organisations in the local community, a range of solutions and initiatives were proposed.

In the present study, it was of great importance to include citizens with type 2 diabetes, cardiovascular disease and/or severe obesity in a process and a range of workshops that gave space to their voice and could encourage a shared understanding and sense of ownership. Our experience and dialogue with the citizens align with the results from the post-workshop questionnaires, which conclude that participants found the project relevant, felt that their voices had been heard, and were generally motivated to continue participation in the process. Most of the citizens participated in all three workshops and expressed commitment by actively taking part in the discussions. Their engagement was essential for the prototyping process, where participation of citizen representatives, as well as other local stakeholders, was a necessity for developing the four different interventions (see Additional file 2 for a description).

### A conceptual model

A secondary aim of the project was to develop a conceptual model for co-creation. In the literature, one finds a plethora of models, frameworks, and approaches to guide community health promotion [31] and often, the distinction between models, frameworks and approaches is unclear as they are used interchangeably. In line with Nilsen, we define a model as one that ‘…typically involves a deliberate simplification of a phenomenon or a specific aspect of a phenomenon’ [32]. The conceptual model depicted in Fig. 2 is based on the learnings from the project and draws upon theories of health promotion and co-creation. The key insights are that achieving outcomes and building capacity at the local level are facilitated by collaborative decision making, a shared understanding of objectives and goals, local planning and action, creating and maintaining trust between stakeholders, and the presence of knowledgeable and trained staff [11, 31]. Both a project leader from the research institution and a local coordinator were important links between and the researchers and between the stakeholders involved in the project. Across all three phases, it was important that the ideas and interventions developed during the process made sense for all stakeholders and added value to both the citizens in the target group, the municipality, and the civil society organisations. Reflecting on the scalability of the model raises questions on how to apply the model in other contexts and in which ways it can and should be adapted to new contexts. Future research can investigate if this conceptual model, developed in a local Danish context, is likely to be applicable and generalisable across sectors and settings. Central to these considerations about scalability is an awareness of the mechanisms of action in what has been called ‘co’approaches [11]. These mechanisms have been described “…as developing a shared understanding, identifying, and meeting needs, giving everyone a voice and sense of ownership, and creating trust and confidence.” [11]. Implementation of the model in new contexts should support a process and a range of activities that ensure the presence of the above mechanisms. However, the heterogeneity of Danish municipalities, both in terms of organization, demography and the political landscape, makes the implementation of the model far from easy. The greatest challenge may be economic constraints, which again could enhance socioeconomic inequality, as health promotion that is not mandatory may only be prioritised by the more prosperous municipalities. Another reflection about scalability of the model concerns ‘real-world’ feasibility, i.e., if the co-creation process outlined could be conducted in practice without the involvement of a research institution. In our experience, it gave legitimacy to the project internally in the municipality that it was initiated and led by a research institution, but a clear answer to this question is not obvious. In regard to sustainability of the project, we have experienced some of the same barriers that are mentioned in the literature on sustainability of interventions, specifically: (1) lack of funding and material resource availability, and (2) lack of documentation of effectiveness and value of the program [6, 33–36]. At the present time (August 2023),) the process of implementing the project in the municipality is still ongoing.

### Strengths and limitations

Considerable strengths of this study are (1) as a result of collaboration between many different stakeholders in the local community, we identified linking interventions that were subsequently implemented, and (2) a consensus was obtained among these stakeholders to proceed to a phase of small-scale testing of the interventions. It proved to be of great value that the project had received sufficient funding to support the testing of local linking activities, generated in the workshops and working groups. Moreover, stakeholder mobilisation and engagements are central tenets in participatory research [27], and it can be difficult to disentangle analytically which preparatory activities in the local community that contributed to these factors. However, we anticipate that the participatory processes—including the explorative work in the local community and the workshops—stimulated the receptiveness of the municipality and the local community towards engaging in the project and allocating resources to it. In that respect, community acceptance is mentioned in the literature to improve participation, data quality, and uptake of results [37].

The dark side of co-production has also been subjected to debate [38, 39] and it has been emphasised that co-production is not straightforward, but rather time-consuming, i.e. taking longer time than traditional research [38]. This was also a practical learning from the present study where the explorative work, the workshops, and working groups in the community all in all required almost 12 months of work. However, we are still convinced that a partnership and co-creative approach was the right choice for this project. Ensuring stakeholder engagement—a guiding principle in participatory research and in this project—would have been difficult to achieve using a more traditional top-down approach.

Another limitation of co-production is the well-established fact that participants in co-creative workshops are not necessarily representative of the general population for whom the interventions are designed [40, 41]. In our case, we had problems recruiting the most vulnerable citizens with either type 2 diabetes, cardiovascular disease and/or severe obesity. Thus, we sought, during the workshops, to take this into account by asking the participants to incorporate in the discussion the perspectives of vulnerable relatives, neighbours, friends, etc. The number of participants in the workshops varied from 10 in workshop 3 to 30 participants in workshop 4 and 32 participants in workshop 5. The retention of participants across workshops was high. Time was invested in building trust with the participants and a sense of ownership was encouraged, by emphasising the importance of the participants’ contribution to the project.

## Conclusion

This study documents lessons learnt in a co-creation process to link a public health care institution in the municipality with civil society organisations in the local community. Furthermore, the study has in detail defined and implemented productive co-creative processes and documented the various phases in a conceptual model that we anticipate can be used by others pursuing similar endeavours.

### Supplementary Information

**﻿Additional file 1:**  The participants’ satisfaction: Results from the post-workshop evaluation questionnaires.

**﻿Additional file 2:** Description of the four co-created interventions.

## Data Availability

Data sharing is not applicable to this article as no datasets were generated or analysed during the current study. Interview guides and scripts used for the workshops are available from the corresponding author on reasonable request.

## Abbreviations

CFAS: The Centre for Physical Activity Research
TIPL: The Intersectoral Prevention Laboratory
SDCS: Steno Diabetes Center Sjaelland
WS: Workshop
