# Supplementary material for: Co-creating active communities: processes and outcomes of linking public rehabilitation programs with civic engagement for active living in a Danish municipality
Source: Res Involv Engagem. 2023 Sep 14;9:83. doi: 10.1186/s40900-023-00495-6 (PMC10503125; doi:10.1186/s40900-023-00495-6)
Supplement: Supplementary file 1 — ﻿Additional file 1: The participants’ satisfaction: Results from the post-workshop evaluation questionnaires. [file 40900_2023_495_MOESM1_ESM.docx]

Additional file 1

Questionnaire data on participant satisfaction with the workshops. WS = workshop.


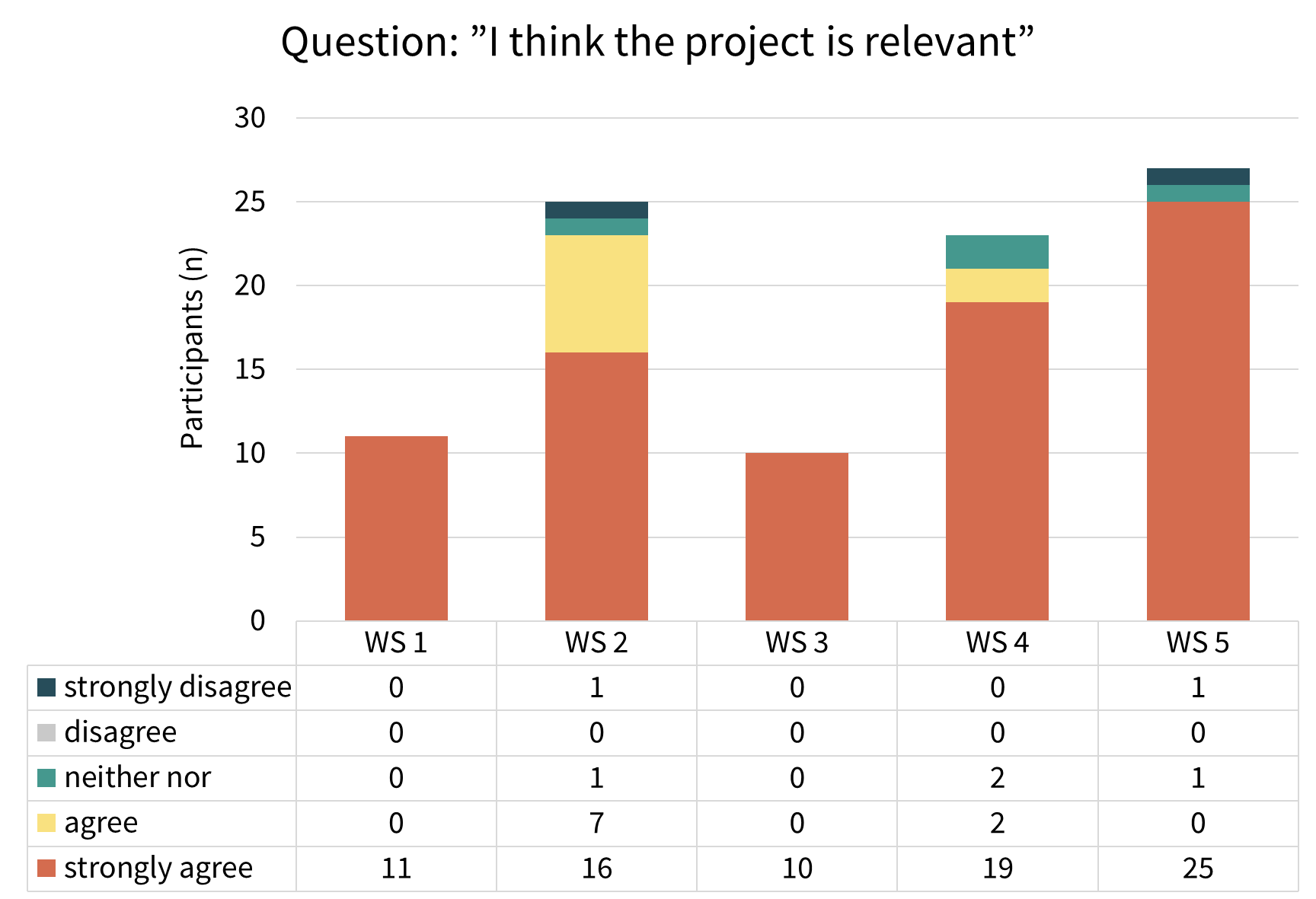


Figure 4a: Question: “I think the project is relevant”


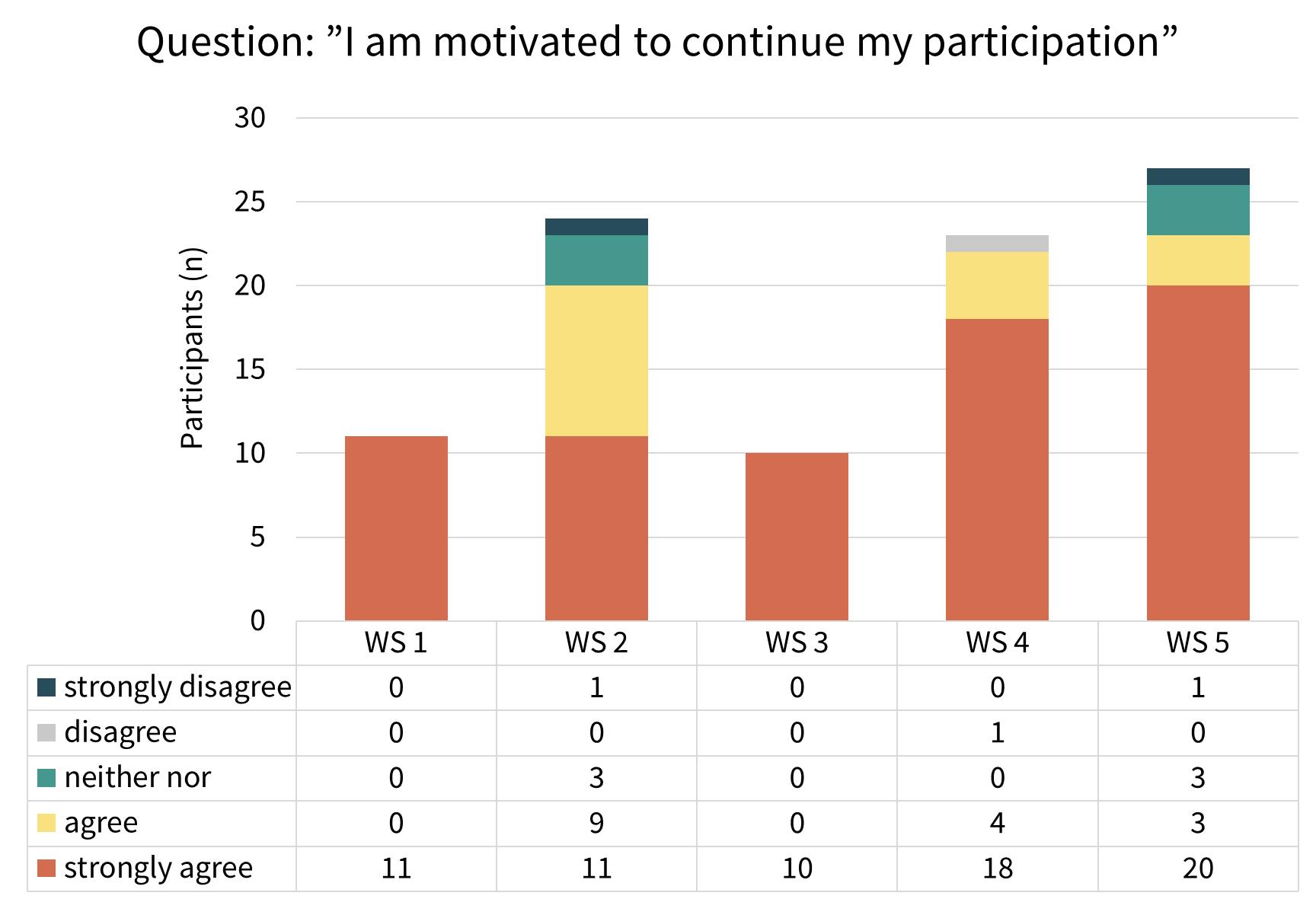


Figure 4b: Question: “I am motivated to continue my participation”


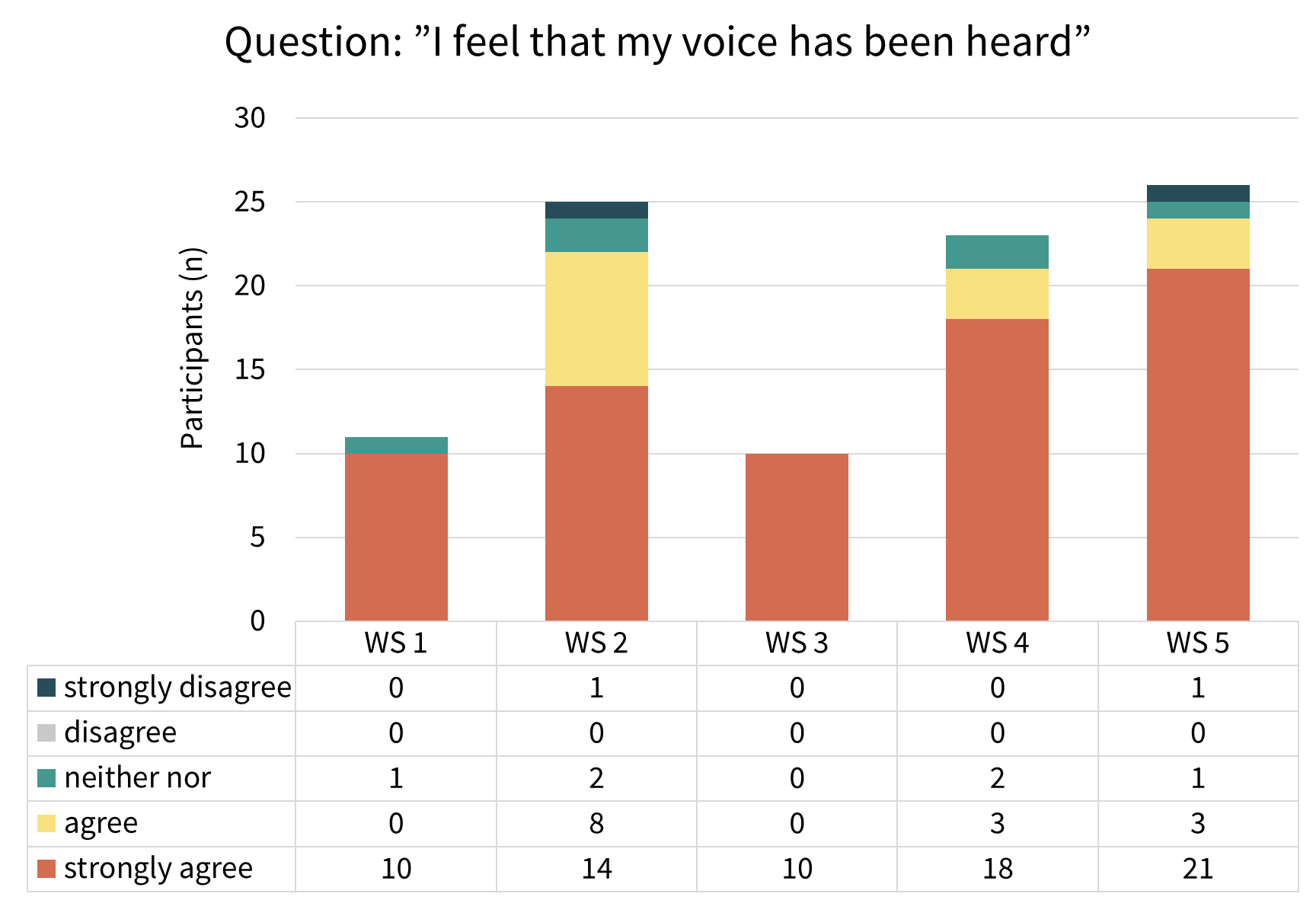


Figure 4c: Question: “I feel that my voice has been heard”
