# Supplementary material for: Co-creating active communities: processes and outcomes of linking public rehabilitation programs with civic engagement for active living in a Danish municipality
Source: Res Involv Engagem. 2023 Sep 14;9:83. doi: 10.1186/s40900-023-00495-6 (PMC10503125; doi:10.1186/s40900-023-00495-6)
Supplement: Supplementary file 2 — ﻿Additional file 2: Description of the four co-created interventions. [file 40900_2023_495_MOESM2_ESM.docx]

Additional file 2

*Description of the four interventions developed in the prototyping phase*

The first intervention to be defined was “Strengthened information and knowledge exchange” (Digital platform and dialogue forum). In this working group consensus was obtained to proceed with the elaboration and creation of a digital platform in the form of a website hosted by the municipality giving an overview of activities and exercise opportunities relevant for the citizens in the target group. The intended audience for the website were health professionals in the municipality, whom together with the citizens are provided with an overview of relevant physical activities in the municipality. Both the citizens and health professionals were involved in the design of the website.

The second intervention was named “Co-created activities and exercise opportunities”. Based on the needs of the local sports and exercise associations, as expressed on the workshops (they needed more knowledge about the citizens in the target group), the project leader conducted focus group interviews with the health professionals in the municipality. The health professionals reflected on these citizens as not being used to participate in exercise in local sports and exercise associations. Adding to this the health professionals stated that focus should not be on the citizens’ diseases (type 2 diabetes, heart diseases and obesity related complications). These reflections, including inspiration on how to adjust the exercise activities to better suit the citizens, were written down in a pamphlet and sent to the local sports and exercise associations. In a subsequent network meeting between local sports and exercise associations and health professionals, co-created activities and exercise opportunities tailored for the citizens in the target group, were debated. An activity is now being developed where a sport association together with the local diabetes association and a municipal health professional hosts theme meetings and events with focus on type 2 diabetes.

The third intervention was named “Integrated activities between sports associations, municipality and citizens with a special focus on utilizing the capacity at exercise facilities and creating a visiting program”. At first, we invited the manager of the municipal exercise facilities for at discussion of better use of the exercise facilities. This led to several activities, that were tested. For instance, a public rehabilitation team ‘the lifestyle team’, decided jointly to continue in a fitness association. The link worker helped to facilitate this and made sure that there was a volunteer present in the fitness association. Participants prioritised to go together to the same activity rather than finding an offer close to their home. Thus the ‘lifestyle team’ continued together in the association and the same volunteer met them at regular times for the next 2-3 weeks until they felt confident at the site. Another tested activity was moving a diabetes team out to an exercise association. The first three training sessions took place at the public health care institution and the last three sessions took place in an exercise association that wants to make an effort for citizens with type-2 diabetes. The health professional in the municipality accompanied and oversaw the teaching, but the training was provided by the association. A final intervention that is currently being tested is to invite an exercise association's instructor to the health center to conduct the teaching on the public rehabilitation team. The instructor came up with new exercises and inspired the citizens to the type of exercise she presented.

The fourth intervention was named “Upgrading municipal resources to achieve better transitions between public rehabilitation and exercise opportunities by employing a link worker”. There was broad agreement on the need for upgrading municipal resources and internal communication, and the need to employ a link worker in the municipality. Funds were allocated in the project for a temporarily (one year) employment of a link worker. Everyone agreed that even though there could not be funds to permanently anchor the position after the project period, it was now possible to try out some actions/activities that would not otherwise have been possible. The job position entails being a contact person and coordinator between the municipality and the different physical activities opportunities in civil society. Furthermore, the link worker’s job is to support citizens, that experience difficulties with being physically active after the end of public rehabilitation. The link worker was employed on the 1^st^ of March 2022. In the work with defining and deciding on the new link worker position, various actors were involved in defining success criteria for the position. Relevant municipal policy committees were briefed and invited to provide input on their wishes for what a link worker should achieve. In this way, increasing the possibility of permanently anchoring the link worker position, if the link worker meets the set success criteria, were anticipated.
